# Targeting cGAS-STING-macrophage axis: a novel therapeutic horizon for renal fibrosis

**DOI:** 10.3389/fimmu.2026.1809851

**Published:** 2026-04-22

**Authors:** Qi Wei, Kui Zhao, Fuyun Jia, Jing Zhou, Tianrui Liu, Shengwei Gao

**Affiliations:** 1 First Teaching Hospital of Tianjin University of Traditional Chinese Medicine, Tianjin, China; 2 National Clinical Research Center for Chinese Medicine, Tianjin, China; 3 Second Affiliated Hospital of Tianjin University of Traditional Chinese Medicine, Tianjin, China

**Keywords:** cGAS-STING, inflammation, macrophages, renal fibrosis, targeted therapy

## Abstract

Renal fibrosis, a pivotal pathological hallmark of chronic kidney disease (CKD), arises from persistent inflammatory responses and extracellular matrix (ECM) deposition. Emerging evidence indicates the cyclic GMP-AMP synthase-stimulator of interferon genes (cGAS-STING) signaling pathway has exerted a crucial influence on the progression of renal fibrosis. This pathway exacerbates the release of inflammatory factors and ECM deposition through reprograming the polarization state of macrophages, thereby driving renal fibrosis. This review delineates the regulatory role of the cGAS-STING signaling pathway in macrophage-related renal fibrosis and critically evaluates emerging innovative strategies targeting this pathway, including small molecule inhibitors, nanocarrier-based delivery systems, and gene editing technologies. However, current research still faces certain limitations, including the complexity of molecular mechanisms, differences in research results, and challenges in clinical translation. By synthesizing recent advances in cGAS-STING-mediated macrophage reprogramming for renal fibrosis intervention, this review aims to provide a foundation for precise therapeutic development.

## Highlights

- Elucidates the central role of the cGAS-STING pathway in driving renal fibrosis.
- Reveals the dual regulatory role of macrophage polarization in renal fibrosis.
- This review critically evaluates emerging therapeutic strategies targeting this pathway (e.g., small molecule inhibitors, nanocarrier-based delivery systems, gene editing technologies).

## 1 Overview of renal fibrosis and its immuno-inflammatory landscape

Chronic kidney disease (CKD) is defined by a sustained decrease in glomerular filtration rate (GFR) or elevated urinary albumin excretion (1). Its progression to end-stage renal disease (ESRD) frequently culminates in renal failure, severely impairing patient quality of life and ranking among the top ten contributors to global disease burden (1, 2). From 1990 to 2016, worldwide CKD incidence, mortality rate, and disability-adjusted life years rose by 89%, 98%, and 62%, respectively, underscoring a critical public health challenge (3). Advanced CKD is often characterized by irreversible kidney fibrosis, manifested as glomerulosclerosis, tubular atrophy, and interstitial fibrosis (2). Given the high incidence and mortality rate of CKD, the irreversibility of kidney fibrosis, and limited therapeutic options, in-depth research on its mechanisms is imperative. Inflammatory cell infiltration and fibroblast activation drive renal fibrosis (4). During this process, the polarization state of macrophages serves as a key regulatory node. Classically activated M1-type macrophages exacerbate tissue damage via pro-inflammatory cytokines such as TNF-α, IL-12, and IL-6 (5). While excessive M2 polarization promotes fibroblast activation through mediators such as IL-22 and transforming growth factor-β (TGF-β) (6, 7). Consequently, reprogramming macrophage polarization is regarded as a promising therapeutic strategy.

Emerging evidence positions the cyclic GMP-AMP synthase-stimulator of interferon genes (cGAS-STING) pathway as an upstream regulator of the inflammatory response and extracellular matrix (ECM) deposition in renal fibrosis (8). Cytoplasmic DNA released by damaged renal tubular epithelial cells or other immune cells activates cGAS, catalyzing the synthesis of 2′3′-cyclic guanosine monophosphate-adenosine monophosphate (2′3′-cGAMP) (9). This second messenger induces STING translocation from the endoplasmic reticulum (ER) to the Golgi apparatus. In the Golgi apparatus, STING oligomers recruit and activate TANK-binding kinase 1 (TBK1) and the inhibitor of nuclear factor κB kinase complex (IKK complex), phosphorylating interferon regulatory factor 3 (IRF3) and activating nuclear factor κB (NF-κB). These transcription factors cooperatively induce pro-inflammatory factors (e.g., IL-6, TNF-α, and IL-1β) and pro-fibrotic factors (e.g., TGF-β and IFN-I), driving the inflammatory state and the massive deposition of collagen and fibronectin (10, 11). Notably, the cGAS-STING pathway not only promotes fibroblast transformation but also induces Macrophage-to-Myofibroblast Transition (MMT), establishing a fibrosis-amplifying loop (8, 12). The cGAS-STING signaling pathway has a crucial role in renal inflammation and fibrosis, providing a new and important idea for the development of targeted interventions to treat renal fibrosis.

In this review, we delineate how the cGAS-STING signaling pathway governs macrophage polarization and its consequential impact on renal fibrosis. We emphasize therapeutic opportunities for targeting this pathway (e.g., cGAS inhibitors, STING inhibitors, TBK1 inhibitors) to reshape macrophage polarization and disrupt fibrotic progression. Moreover, we also expound the strategies and prospects of using other emerging technologies (nanocarriers, gene editing technology) to treat renal fibrosis through precisely targeting the cGAS-STING signaling pathway in macrophages. Therefore, in-depth exploration of the interaction between the cGAS-STING pathway and macrophage polarization not only contributes to revealing the core molecular mechanism of renal fibrosis, but also may establish a theoretical foundation for the development of anti-fibrotic therapies.

## 2 The main characteristics and mechanisms of renal fibrosis

**Figure 1:**
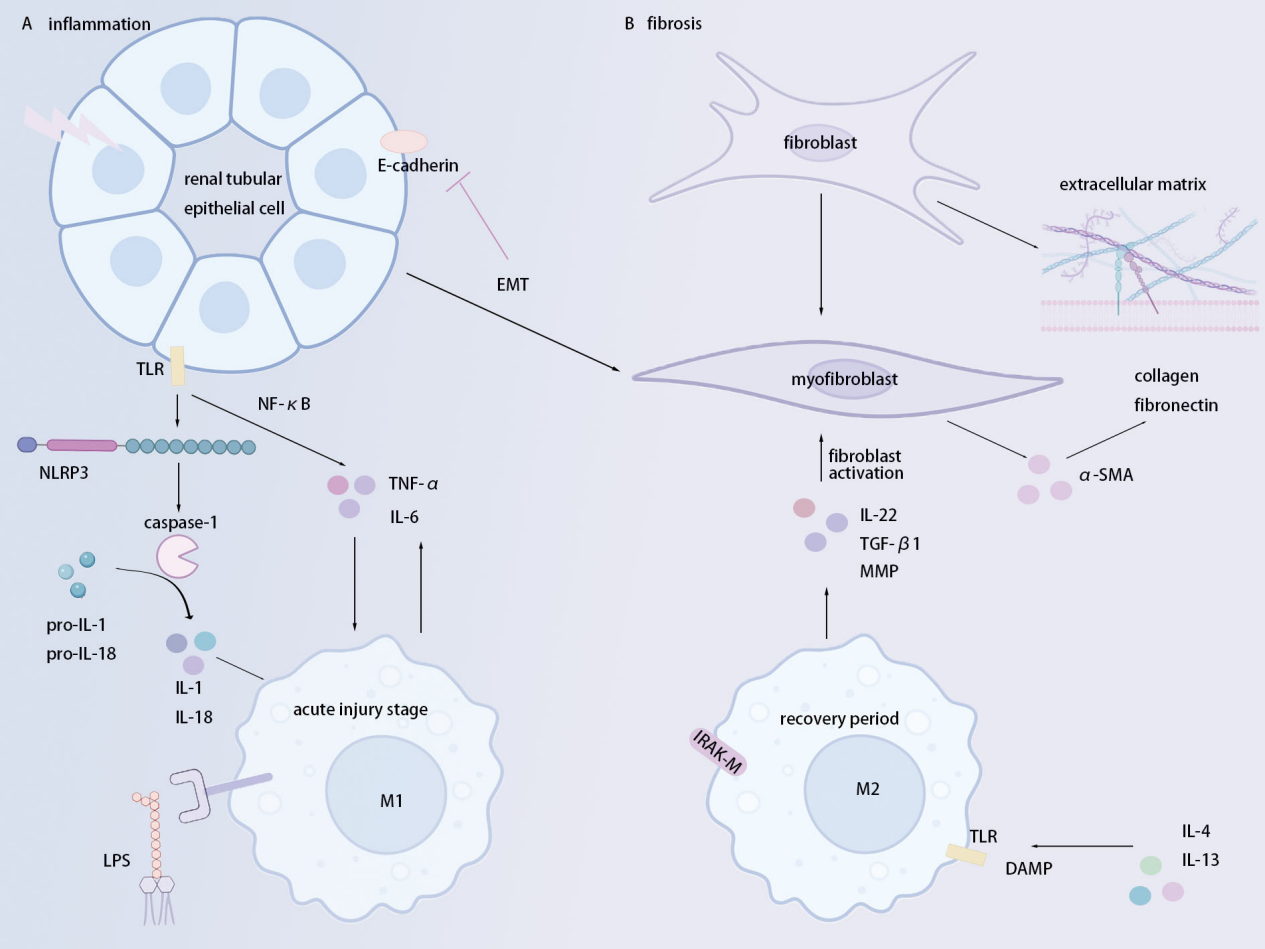
Key pathogenic mechanisms driving inflammation and fibrosis in renal fibrosis. **(a)** Inflammation phase (Acute injury): Renal tubular epithelial cell (RTEC) injury triggers the TLR/NF-κB pathway and NLRP3 inflammasome activation, leading to caspase-1-dependent maturation and release of potent pro-inflammatory cytokines (IL-1β, IL-18, TNF-α, IL-6). This inflammatory cascade promotes M1 macrophage polarization, establishing a self-amplifying cycle that exacerbates tissue damage. **(b)** Fibrosis phase (Repair/Recovery): Repair state drives macrophage polarization toward the M2 phenotype and fibroblast activation into matrix-producing myofibroblasts. Key mediators include TGF-β1 (potent fibrogenic driver), IL-4, IL-13, and IL-22. This results in excessive extracellular matrix (ECM) deposition (collagen, fibronectin) and α-SMA expression, culminating in renal fibrosis.

Renal fibrosis culminates in irreversible renal structural damage and progressive decline in renal function, eventually advancing to ESRD. This progression manifests as glomerular sclerosis, renal tubular atrophy, and renal interstitial fibrosis (2, 13). Renal tubular epithelial cells (RTECs) are central to both kidney injury response and fibrosis repair. While RTECs can self-repair following obstruction, ischemia, or toxin exposure, persistent or exaggerated repair drives interstitial fibrosis and ESRD (13). The inflammatory response induced by injury plays a crucial role in the fibrosis process. (**Figure 1**) Kidney injury can activate inflammation in RTECs, fibroblasts, and macrophages (4). RTECs express Toll-like receptors (TLRs) that trigger NF-κB signaling, amplifying local inflammation via TNF-α and IL-6 production (14). Concurrently, the activation of the NLRP3 inflammasome (NOD-, LRR-, and pyrin domain-containing protein 3) in RTECs is another key mechanism for the inflammatory response (15). Once activated, the NLRP3 inflammasome recruits caspase-1, catalyzing the maturation of pro-IL-1β and pro-IL-18 to exacerbate the cascade of inflammation (16).

It is worth noting that the sustained inflammation can induce epithelial-mesenchymal transition (EMT) in RTECs, considered an important driving mechanism of renal fibrosis. EMT loses epithelial cell markers (e.g., E-cadherin) and acquires a mesenchymal phenotype, showing increased α-smooth muscle actin (α-SMA) expression (17). This transition confers myofibroblast-like properties, enhancing cell migration and invasion abilities, thus promoting the deposition of ECM (17). Therefore, the injury of RTECs not only triggers the inflammatory response but also exacerbates the process of renal fibrosis through EMT. Fibroblasts are a type of mesenchymal cell with a spindle-shaped morphology (17). Their abnormal activation is pivotal for the abnormal progression of renal fibrosis. Fibroblasts mainly synthesize ECM components such as collagen and fibronectin. Activated by inflammatory factors, growth factors, hypoxia, and mechanical stress, they synthesize collagen and fibronectin to initially repair renal tissue damage (18). However, aberrant differentiation into α-SMA+ myofibroblasts perpetuates pathological ECM accumulation in scar tissue (4, 18, 19). Overall, inflammation, RTEC-driven EMT, and fibroblast-myofibroblast activation converge to fuel renal fibrosis through unchecked ECM deposition.

## 3 cGAS-STING signaling pathway and its role in immune surveillance

### 3.1 Structure of cGAS-STING

cGAS, a nucleotide transferase (NTase) family member, comprises an NTase domain and two DNA-binding domains (20). In the resting state, cGAS exists in an auto-inhibited conformation. When activated, the two cGAS molecules form a 2:2 cGAS-DNA dimer by binding to two DNA strands (21). This dimerization process promotes the conversion of cGAS from an inactive state to a highly active state. Subsequently, the activated cGAS catalyzes the synthesis of the second messenger 2′3′-cGAMP from ATP and GTP (21). STING, a membrane-resident protein localized in the ER (22), contains a short cytoplasmic region at the N-terminus, four transmembrane helices, a cytoplasmic ligand-binding domain (LBD), and a disordered C-terminal tail (CTT) (23). STING constitutively dimerizes via its LBD. When 2′3′-cGAMP inserts into the central gap of the STING dimer, it induces a conformational change in the LBD (24), exposing the previously hidden CTT and creating a binding groove for TBK1 (25). This recruits the TBK1 dimer to trigger downstream immune signal transduction (25).

### 3.2 Activation and signal transduction of the cGAS-STING pathway

Cytosolic DNA serves as a critical immune danger signal, detected by cGAS. Emerging evidence indicates that both exogenous DNA from microbial infections and endogenous DNA released through DNA damage, mitochondrial damage, or cell death activate cGAS, triggering the immune response (9). In addition to cytosolic DNA, nuclear DNA (nDNA) is also considered critical for activating cGAS. For instance, under pathological conditions such as oxidative stress, mechanical strain, or genomic instability, the integrity of the nuclear envelope may be compromised, leading to leakage of nuclear DNA into the cytoplasm (26). Concurrently, nDNA damage leads to increased release of DNA fragments, thereby activating the DNA sensor cGAS (26). Upon binding to cytosolic DNA, cGAS undergoes conformational changes and catalyzes the synthesis of second messengers 2′3′-cGAMP from ATP and GTP (9).

Subsequently, 2′3′-cGAMP binds to the STING dimer on the ER, inducing its conformational changes and oligomerization. Oligomeric STING recruits ER export protein 1 (STEPP), promoting accumulation of phosphatidylinositol 3-phosphate (PI3P). PI3P mediates ER membrane curvature and leads to the formation of COPII vesicles (27). This process enables STING trafficking from the ER through the ER-Golgi intermediate compartment (ERGIC) to the Golgi apparatus (27). On the Golgi membrane, STING oligomers recruit and activate TBK1 and the IKK complex (10). Activated TBK1 phosphorylates Ser366 with its STING-CTT, providing a binding site for interferon regulatory factor 3 (IRF3) (10). Subsequent TBK1-mediated phosphorylation of IRF3 drives its dimerization, nuclear translocation, and induction of type I interferons (IFN-I) (10). Concurrently, the IKK complex activates NF-κB, leading to its nuclear entry and transcription of pro-inflammatory cytokines, including TNF-α and IL-6 (11). In summary, the cGAS-STING signaling pathway acts as a central hub of innate immunity, integrating signals from exogenous and endogenous cytosolic DNA to orchestrate inflammatory cytokine and interferon production.

## 4 cGAS-STING signaling pathway in renal fibrosis

### 4.1 The effect of the cGAS-STING signaling pathway on inflammation

**Figure 2:**
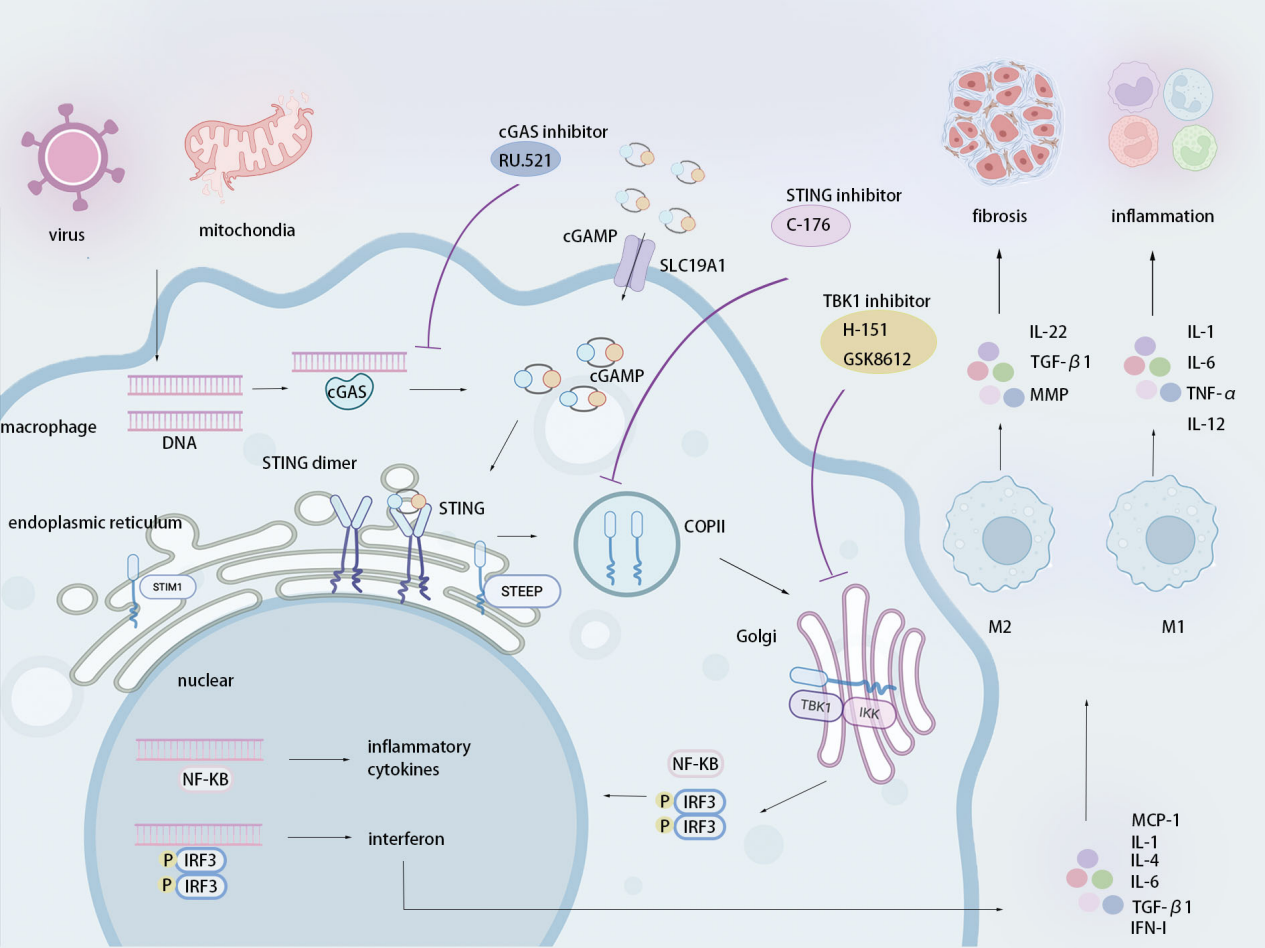
The cGAS-STING signaling pathway mediates macrophage polarization.

Renal inflammation, a key precursor event in the progression of renal fibrosis, is characterized by elevated expression of pro-inflammatory cytokines, including IL-6, TNF-α, and IL-1β. Damaged RTECs amplify the release of pro-inflammatory cytokines by activating the NF-κB pathway and the NLRP3 inflammasome (14, 16). (**Figure 2**) Emerging evidence reveals the cGAS-STING signaling pathway as a pivotal regulator of this inflammatory cascade. Specifically, upon recognizing mitochondrial DNA (mtDNA) released during mitochondrial damage, cGAS triggers the downstream signaling pathway of STING. Activated STING recruits the IKK complex, leading to phosphorylation of nuclear factor-κB inhibitor and subsequent nuclear translocation of NF-κB, which upregulates the transcription of pro-inflammatory cytokines (e.g., TNF-α and IL-6) (28, 29). For example, in renal injury models driven by mitochondrial transcription factor A (TFAM) deficiency, STING knockout can significantly reduce NF-κB activation and the expression of downstream pro-inflammatory factors, confirming STING’s role in inflammation via NF-κB (30).

Endogenous or exogenous DNA is recognized by cGAS, which catalyses the synthesis of the second messenger cGAMP. The binding of cGAMP to STING dimers on the ER induces STING oligomerization. Activated STING oligomers are transported from the ER via COPII-coated vesicles to the Golgi membrane. At the Golgi, activated STING recruits and activates the kinases TBK1 and the IKK complex. TBK1 and IKK then phosphorylate the transcription factors IRF3 and NF-κB. Phosphorylated IRF3 forms dimers and, together with activated NF-κB, translocates to the nucleus. This induces the expression of various inflammatory factors. These factors promote M1 macrophage polarization, amplifying the inflammatory response. Conversely, cytokines, including TGF-β1 and IL-22, induce M2 macrophage polarization, which promotes the deposition of ECM components such as collagen and fibronectin, contributing to fibrosis.

Furthermore, the cGAS-STING signaling pathway regulates the NLRP3 inflammasome activation. For instance, in Pseudomonas aeruginosa-infected mice lacking the autophagy-related protein 5 (ATG5), the massive mtDNA release in macrophages activated the cGAS-STING signaling pathway, triggering the activation of the NLRP3 inflammasome and the inflammatory cascade reaction (31). It is worth noting that oxidized mtDNA (ox-mtDNA) has been identified as a direct NLRP3 inflammasome activator (32–34). The activated NLRP3 inflammasome cleaves pro-IL-1β via caspase-1, further amplifying NF-κB signaling and pro-inflammatory cytokines (e.g., IL-6, TNF-α, and IL-1β) (34). In conclusion, the activation of NF-κB and the NLRP3 inflammasome are central to establishing the inflammatory microenvironment that drives early renal fibrosis. The cGAS-STING pathway serves as an indispensable upstream regulator, propagating inflammation and fibrotic progression through these key effectors.

### 4.2 cGAS-STING signaling pathway in fibrosis

During the occurrence and development of renal fibrosis, fibroblast dysregulation is regarded as a central driver of it. The cGAS-STING signaling pathway contributes critically to this process through dual mechanisms. (**Figure 2**) First, it directly promotes the activation of fibroblasts and myofibroblasts. Second, it significantly enhances ECM deposition via a range of pro-inflammatory and pro-fibrotic cytokines (8, 35). Evidence from unilateral ureteral obstruction (UUO) models demonstrated that STING deletion not only significantly reduced the number of α-SMA+ myofibroblasts, but also downregulated the expression levels of total collagen and ECM components, including fibronectin and type I collagen (8). This establishes cGAS-STING activation as essential for α-SMA+ myofibroblast expansion and pathological ECM accumulation.

Furthermore, the activation of the cGAS-STING signaling pathway amplifies the ECM infiltration by upregulating cytokines such as TGF-β, IFN-I, IL-6, and TNF-α). TGF-β, a master fibrogenic regulator, can stimulate activated macrophages to secrete pro-fibrotic cytokines and induce their transition to a myofibroblast phenotype (36, 37). Similarly, IFN-I also participated in the abnormal accumulation of collagen (38). For instance, in systemic sclerosis, elevated IFN-I exacerbates ECM deposition by promoting fibroblast activation and potentiating TGF-β signaling, further aggravating ECM deposition (39). Thus, the cGAS-STING signaling pathway accelerates kidney fibrosis through a dual mechanism of directly activating matrix-producing myofibroblasts and amplifying profibrotic cytokine networks.

In addition to the influence of fibroblasts, metabolism also plays a significant role in renal fibrosis. In the fibrotic kidney, injured RTECs undergo a profound metabolic shift characterized by enhanced glycolysis. This increased glycolysis is associated with mitochondrial dysfunction. For example, in a UUO mouse model of renal fibrosis, pyruvate carboxylase deficiency in RTECs leads to impaired mitochondrial structure and function, resulting in increased mtDNA release and subsequent activation of the cGAS-STING pathway. Activation of this pathway further enhances glycolysis by regulating hypoxia-inducible factor HIF-1α (40). The increased expression of glycolytic enzymes, including HK2 and PKM2, exacerbates interstitial fibrosis and impairs renal function (35). Therefore, RTECs may promote renal fibrosis by activating the cGAS-STING pathway, which upregulates glycolytic metabolism.

## 5 Macrophages in renal fibrosis

### 5.1 The dual role of macrophages in kidney fibrosis

Macrophages, key innate immune cells maintaining tissue homeostasis and host defense, can dynamically polarize in response to microenvironmental cues, adopting pro-inflammatory (M1) or pro-healing (M2) phenotypes. In the early phase of acute kidney injury (AKI), the local inflammatory microenvironment drives macrophages to polarize toward the M1 type. (**Figure 1**) Specifically, damaged RTECs and infiltrating neutrophils release a large amount of reactive oxygen species (ROS), establishing a potent oxidative stress milieu (5). Concurrently, CD11c+ kidney dendritic cells secrete abundant TNF-α and, via binding to the sphingosine-1-phosphate receptor-3 (S1P3) of natural killer T (NKT) cells, amplify interferon-γ (IFN-γ) production (41). These cytokines activate resident macrophages and facilitate the differentiation of infiltrating monocytes into the pro-inflammatory M1 phenotype. M1-type macrophages secrete abundant pro-inflammatory cytokines, such as TNF-α, IL-12, and IL-6, significantly exacerbating renal inflammation (5).

However, once the recovery period begins, the renal microenvironment shifts from a pro-inflammatory state to a state that promotes repair and fibrosis. Macrophages consequently repolarize from the pro-inflammatory M1 type to the pro-repair M2 type. Notably, IFN-γ administration elicits divergent effects: during injury, it enhances M1 polarization, whereas in recovery (e.g., post-day 3 of ischemia-reperfusion), it drives M2 expression (42). This underscores the microenvironment’s decisive role in macrophage plasticity. During the recovery phase, M2 polarization is selectively activated by IL-4/IL-13 in the microenvironment. Upon recognizing damage-associated molecular patterns (DAMPs) via TLR4, M2 macrophages upregulate the production of interleukin-22 (IL-22) through downstream signaling, promoting the proliferation of RTECs (6). They also secrete TGF-β1 to sustain myofibroblast survival and modulate matrix metalloproteinases (MMPs), maintaining the activation of myofibroblasts and mediating ECM homeostasis (7). Furthermore, the IL-1 receptor-associated kinase-M (IRAK-M) of M2 inhibits TLR/IL-1R signaling transmission, resolving kidney inflammation (43). Critically, RTEC-derived macrophage colony-stimulating factor (M-CSF) enhances M2-mediated repair, establishing a reciprocal repair axis (44, 45). In summary, macrophages exhibit highly environment-dependent phenotypic transitions: M1 dominance propagates early injury, while M2 polarization, via IL-22, TGF-β1, and IRAK-M, orchestrates tissue repair and inflammation resolution during recovery.

### 5.2 Macrophage polarization and renal fibrosis

In renal fibrosis, macrophage polarization is critically linked to inflammation and fibrosis, making its targeted regulation an emerging therapeutic strategy. Aberrant activation of the classical Wnt/β-catenin signaling pathway directly amplifies the pro-fibrotic potential of macrophages (46). Specifically, Wnt3a exacerbates the synergistic effect of IL-4 or TGF-β1, potentiating the polarization of macrophages to the M2 phenotype. Subsequent β-catenin nuclear translocation and binding to T-cell factor (TCF)/lymph enhancer binding factor upregulates pro-fibrotic genes (e.g., Snail and PAI-1), accelerating collagen deposition (46, 47). The knockout of β-catenin in macrophages significantly reduces the infiltration of M2-type macrophages and α-SMA+ myofibroblasts in the kidneys, confirming its central role in driving M2 polarization and inducing the activation of myofibroblasts (46).

Twist1, a member of the basic helix-loop-helix transcription factor family, has been confirmed to be critically related to fibrosis (48). In RTECs, Twist1 promotes M2 polarization in rRTECs by upregulating galectin-3, which induces IL-4 secretion (49). Emerging studies proved that galectin-3 is mainly secreted by macrophages and can further activate various pro-fibrotic factors, promoting collagen deposition and fibroblast proliferation (50). Pharmacological inhibition of Twist1 (e.g., with metformin) can reduce M2 infiltration in the kidney, supporting Twist1/Galectin-3 axis as a viable anti-fibrotic target (51).

G protein-coupled estrogen receptor 1 (GPER1), a novel estrogen receptor, plays a significant role in modulating macrophage polarization and renal fibrosis (52). Evidence is mounting that GPER1 agonists can downregulate the expression of TLR4 in macrophages and inhibit the activation of NF-κB induced by LPS, significantly suppressing the release of M1-related inflammatory mediators (53, 54). Additionally, the activation of GPER1 also interferes with the profibrotic effect mediated by M2. Activated GPER1 inhibits the transformation of fibroblasts into myofibroblasts and reduces ECM deposition (55). These studies indicate that GPER1 can simultaneously inhibit the inflammatory response mediated by M1 and the fibrotic process mediated by M2, providing a highly promising strategy for targeting macrophages in the treatment of renal fibrosis. In summary, the Wnt/β-catenin, Twist1/Galectin-3 axis, and GPER1 signaling converge on macrophage polarization to drive renal fibrosis. These methods provide a theoretical basis for mitigating inflammatory responses and inhibiting the fibrotic process.

## 6 The cGAS-STING signaling pathway mediates the polarization of macrophages

As discussed above, RTEC injury is a critical initiating event in renal fibrosis. Damaged RTECs activate immune cells through various mechanisms, in which macrophages play a central role. Macrophages exhibit dual functions in renal fibrosis: predominantly M1-type pro-inflammatory activity in the acute phase and M2-type pro-fibrotic activity in the chronic phase. Recent studies have revealed that the cGAS-STING pathway acts as a bridging link between RTEC injury and macrophage reprogramming (35). Under injury-induced stress, RTECs undergo mitochondrial dysfunction and metabolic reprogramming, leading to the release of mtDNA into the cytosol or extracellular space. This mtDNA is recognized by adjacent macrophages, activating the intracellular cGAS-STING signaling cascade, which subsequently promotes the expression of inflammatory cytokines, chemokines, and pro-fibrotic factors.

Of note, the regulation of macrophages by the cGAS-STING pathway is microenvironment-dependent. During the acute injury phase, this pathway amplifies the inflammatory response via inducing M1 polarization, as evidenced in murine models of kidney injury (UUO) and radiation-induced lung injury (RILI). For instance, in a mouse model of kidney injury mediated by UUO, genetic ablation of cGAS/STING significantly reduced F4/80+ macrophage infiltration and pro-inflammatory cytokine expression (e.g., TNF-α, IL-1β, IL-6, monocyte chemoattractant protein-1 (MCP-1), and TGF-β1) (8). This confirms that cGAS/STING is an important factor in inducing M1 polarization. Similarly, in a mouse model of RILI (12.5Gy whole thoracic irradiation), the cGAS-STING pathway significantly activated M1-type macrophage polarization by upregulating CCL2 (MCP-1), exacerbating pro-inflammatory cytokines, and exacerbating tissue damage (56).

In addition to amplifying the inflammatory response, during the repair stage, the cGAS-STING signaling pathway promotes fibrosis through M2-mediated mechanisms. For example, in the UUO model, cGAS/STING knockout attenuated renal expression of collagen I/III and fibronectin in kidneys (8). Additionally, in a mouse model induced by a high-fat diet, STING deficiency reduced collagen deposition and the severity of liver fibrosis (57). These studies consistently establish the pathway’s conserved pro-fibrotic function across organs. In summary, the cGAS-STING signaling pathway orchestrates a dual response. It not only mediates inflammatory responses through M1 polarization but also promotes ECM deposition through M2 polarization. Therefore, targeting inhibition of this pathway simultaneously attenuate M1-driven inflammatory responses and M2-driven fibrosis, providing a potential therapeutic strategy for macrophage polarization–dependent conditions such as kidney fibrosis.

The cGAS-STING pathway triggers inflammatory responses and fibrosis by regulating macrophage polarization. During the acute phase of injury, this pathway preferentially promotes M1 polarization; however, in a chronic fibrotic microenvironment dominated by TGF-β, the same pathway potentiates M2-like pro-fibrotic activity. The biased output of this pathway may be highly dependent on the cellular microenvironment, encompassing both cytokines and cellular metabolism. In the acute phase, the accumulation of various cytokines and LPS mediates STING activation. For instance, in a sepsis model, LPS mediates the activation of the cGAS-STING-IFN-β signaling axis, inducing macrophage polarization toward the M1 phenotype and altering mitochondrial homeostasis and metabolic status (58). Conversely, in a PD-induced encapsulating peritoneal sclerosis model, STING activation in peritoneal mesothelial cells significantly increases the secretion of the macrophage chemokine CCL2, leading to enhanced macrophage infiltration and the formation of pathological adhesions (59). This positive feedback loop amplifies the fibrotic response. These findings suggest that the expression and functional output of the cGAS-STING pathway are highly dependent on the state of the cellular microenvironment.

## 7 Targeting the cGAS-STING signaling pathway to mitigate renal inflammation and fibrosis

After cytoplasmic DNA is recognized by cGAS, cGAS triggers the catalytic formation of the second messenger cGAMP from ATP and GTP. cGAMP activates STING, initiating the TBK1-IRF3/NF-κB signaling cascade, which amplifies renal inflammation and fibrosis. Consequently, pharmacological inhibition of key pathway components, including cGAS, STING, and TBK1, represents a promising therapeutic strategy to disrupt this pathogenic axis.

RU.521, a catalytic site inhibitor of cGAS, occupies the active sites of cGAS and potently suppresses cGAMP synthesis, thereby inhibiting the transduction of the cGAS-STING signaling pathway (60). For example, in murine UUO mouse models, daily RU.521 administration (10mg/kg) significantly reduced the protein levels of inflammatory factors (e.g., TNF-α, IL-1β, IL-6), fibronectin, type I collagen, and α-SMA in renal tissue (**Table 1**) (8). Similarly, in a sepsis mouse model, RU.521 (5mg/kg) also effectively attenuated the expression of cardiac inflammatory factors and improved cardiac function (65). These results indicate the efficacy of RU.521 in curtailing inflammation and collagen deposition via cGAS-STING signaling pathway blockade, providing preclinical evidence for intervention in kidney fibrosis.

**Table 1:** A summary table of drugs, targets and functions related to the cGAS-STING pathway.

Drug/Technology	Target	Model	Associated pathway	Regulated biochemical component	Function	Reference
RU.521	cGAS	murine UUO mouse models	cGAS-STING	TNF-α, IL-1β, IL-6, fibronectin, type I collagen, and α-SMA	Inhibition of the catalytic site of cGAS reduced inflammation and collagen deposition.	(8, 57)
C-176	STING-Cys91	TFAM-deficient renal fibrosis models	STING pathway	TNF-α, IL-1β, IL-6, fibronectin, type I collagen, IFN-I	The inhibition of STING-Cys 91 palmitoylation reduces the occurrence of inflammation and fibrosis.	(58, 59)
H-151	STING-Cys91, TBK1	mouse model of myocardial infarction; mice model of transient middle cerebral artery occlusion	cGAS-STING-TBK1	ECM, IFN-β, TNF-α, IFN-γ, IL-10	The inhibition of the cGAS-STING pathway prevented the progression of kidney inflammation and fibrosis.	(60, 61)
H-151	STING-Cys91, TBK1	folic acid-mediated renal fibrosis models	cGAS-STING-macrophage polarization	macrophage phenotypic switching, fibroblast activation	The cGAS-STING pathway reprograms the function of macrophages.	(12, 60, 61)
adeno-associated virus-delivered VBIT-12 (a VDAC1 inhibitor)	mtDNA	murine UUO mouse models	cGAS-STING	MCP-1, TNF-α, IL-1β, IL-6, collagen I, α-SMA, and fibronectin	The VDAC1 inhibitor inhibited the cGAS-STING pathway.	(62)
Amlexanox (a TBK1 inhibitor)	TBK1	murine UUO mouse models	cGAS-STING	cGAS, STING, pSTING, pTBK1, IFN, ECM	Amlexanox inhibits renal fibrosis through the cGAS-STING pathway.	(63)
lentivirus	STING	mouse models of renal ischemia-reperfusion injury	cGAS-STING	ECM	STING knockdown inhibited the polarization of the cGAS-STING pathway.	(64)
CRISPR-Cas9	cGAS/STING	murine UUO mouse models; mice models with HFD-induced steatosis	cGAS-STING	ECM, TNF-α, IL-1β and IL-6	The knockout of cGAS/STING inhibited the activation of the cGAS-STING pathway.	(8, 55)

CRISPR-Cas9, clustered regularly interspaced short palindromic repeats associated protein 9; TFAM, mitochondrial transcription factor A; UUO, unilateral ureteral obstruction.

Similar to cGAS, the activity of the STING protein is also precisely regulated by various post-translational modifications, notably palmitoylation at Cys91. Specifically, the STING palmitoylation inhibitor C-176 can covalently bind to the Cys91 of STING and block the palmitoylation at this site, inhibiting the downstream signaling pathway of STING (**Table 1**) (61). For example, in a TFAM-deficient renal fibrosis model, C-176 significantly reduced the deposition of collagen and the inflammatory response (30). Moreover, chronic C-176 treatment in *Trex1*-/- mice also suppressed IFN-I production (61). In summary, C-176 inhibits the palmitoylation of STING, validating palmitoylation inhibition as a precise therapeutic approach for targeting the cGAS-STING signaling pathway and intervening in renal fibrosis.

TBK1, a critical downstream protein of cGAS-STING, also plays a pivotal role in inflammation and fibrosis. H-151 blocks the palmitoylation of STING-Cys91 through covalent binding, indirectly impairing the recruitment and phosphorylation of TBK1 (**Table 1**) (66). GSK8612 directly targets the kinase domain of TBK1, blocking its catalytic activity (62). Both strategies attenuate the signal transduction of the STING pathway, exerting anti-inflammatory and anti-fibrotic effects. In folic acid (FA)-induced models, treatment with H-151 or GSK861 significantly reduced the accumulation of myofibroblasts and hindered the differentiation of macrophages into myofibroblasts, thus reducing ECM deposition (12). These results indicate that direct or indirect inhibition of TBK1 can effectively curb the process of renal fibrosis, providing a new strategy for precise targeting of the cGAS-STING signaling pathway in clinical practice. In conclusion, inhibiting the upstream (cGAS, STING) or downstream (TBK1) nodes within the cGAS–STING pathway can effectively alleviate renal inflammation and ECM deposition, providing a robust preclinical foundation for targeting this pathway to treat fibrotic kidney disease.

## 8 Regulating macrophage phenotype through targeting the cGAS-STING signaling pathway

The cGAS-STING signaling pathway, a central regulatory pathway for innate immunity, represents a therapeutic target for modulating macrophage polarization to suppress inflammation and mitigate fibrosis. Selective inhibition of STING with H-151 significantly impedes the transformation of macrophages-to-myofibroblast transdifferentiation by blocking this pathway (12). For instance, in a FA-mediated renal fibrosis model, H-151 attenuated both macrophage phenotypic switching and fibroblast activation, reducing ECM deposition (12). These findings indicate that pharmacological cGAS-STING blockade reprograms macrophage function to ameliorate the renal microenvironment, thereby counteracting inflammation and fibrosis.

Comparable mechanisms operate in cardiac and neurological pathologies. For instance, in a myocardial infarction model, H-151 targeted macrophage cGAS-STING signaling to inhibit the production of IFN-β, suppressing local inflammatory responses and alleviating the occurrence of myocardial cell fibrosis (66). Similarly, after a stroke, the activation of STING mediated the transformation of macrophages to an inflammatory phenotype, exacerbating neuroinflammation. H-151 administration downregulated the expression of pro-inflammatory cytokines such as TNF-α and IFN-γ in macrophages while elevating the expression of anti-inflammatory factor IL-10, subsequently alleviating neuroinflammation and restoring neural function (67). Collectively, H-151-mediated cGAS-STING inhibition reprograms macrophage phenotypes, demonstrating broad anti-inflammatory and anti-fibrotic efficacy across organ systems. This establishes macrophage-centered cGAS-STING targeting as a unifying strategy for fibrosis treatment in diverse diseases.

## 9 Other emerging technologies in targeted therapy

### 9.1 Nanocarrier delivery technology

Nanotechnology is emerging as an important means of intervening in renal fibrosis, with its advantages lying in its precise targeting capabilities and minimal off-target effects. Utilizing nanocarrier technology to target the cGAS-STING signaling pathway provides a novel therapeutic approach for treating renal fibrosis. Voltage-dependent anion channel 1 (VDAC1) is closely related to the cGAS-STING signaling pathway. Specifically, VDAC1 increases the permeability of the mitochondrial outer membrane, facilitating the release of mtDNA into the cytoplasm, and subsequently activating the cGAS-STING signaling to trigger inflammation and fibrosis (64, 68). Consequently, targeting intervention of VDAC1 disrupts cGAS-STING activation, attenuating renal inflammation and fibrosis (69). For instance, in UUO mice models, adeno-associated virus 9 (AAV9)-delivered VBIT-12 (a VDAC1 inhibitor) suppressed cGAS-STING signaling pathway, significantly reducing the expression of pro-inflammatory cytokines (e.g., MCP-1, TNF-α, IL-1β, and IL-6), and fibrosis markers (e.g., collagen I, α-SMA, and fibronectin) (**Table 1**) (69). These findings indicate that inhibitors of the cGAS-STING signaling pathway based on nanocarriers can effectively block the expression of this pathway and achieve precise silencing of it.

Furthermore, the carrier can also intervene in renal fibrosis by targeting the expression of STING. Studies confirmed that viral vector-mediated STING knockdown robustly ameliorates fibrotic phenotypes (70). In a mouse model of renal ischemia-reperfusion injury (IRI), intrarenal delivery of STING-targeted lentivirus markedly reduced collagen deposition, as evidenced by Masson staining (71). This underscores that the STING lentivirus provides a precise tool for precise targeted treatment of renal fibrosis. In summary, nanomaterials targeting the cGAS-STING signaling pathway establish a novel conceptual framework for the targeted treatment of renal fibrosis.

### 9.2 Gene editing technology

Gene editing technologies (such as CRISPR-Cas9) provide novel approaches for directly modulating the cGAS-STING pathway in macrophages. Evidence from murine UUO models demonstrates that genetic ablation of either cGAS or STING attenuates renal inflammation, reduces collagen deposition, and ameliorates fibrosis (8, 57). Importantly, gene-editing technology has further verified that the cGAS-STING pathway is a pivotal driver of renal fibrogenesis. Specifically, in the mouse model with PINK1 knockout achieved through gene editing, Masson’s trichrome staining revealed that PINK1 deficiency promoted mtDNA release and stimulated the cGAS-STING pathway, subsequently mediating the occurrence of renal inflammation and fibrosis (72). Collectively, these gene-editing-based investigations provide compelling evidence supporting the cGAS-STING pathway in macrophages as a therapeutic target, thereby informing a new direction for the treatment of renal fibrosis. Although AAV9-delivered VBIT-12, STING-targeted lentivirus, and PINK1 knockout have shown anti-fibrotic effects in mouse models, these approaches remain at the preclinical proof-of-concept stage. Their long-term safety needs to be evaluated prior to clinical translation.

## 10 Potential adverse effects

While inhibition of the cGAS-STING pathway can ameliorate the progression of renal fibrosis, it may also lead to certain adverse effects. As a crucial component of the human innate immune system, this pathway plays an essential role in viral infections and tumor immunity. For example, in patients with hepatitis B, HBV viral DNA can be recognized by the cGAS-STING pathway, promoting the release of IFN-I and various inflammatory cytokines, thereby blocking viral replication (73). Furthermore, IFN-I not only directly inhibits viral replication but also activates antigen-presenting cells (APCs) and promotes the cross-presentation and activation of CD8+ T cells, thereby suppressing tumor cell growth (74). These studies indicate that this pathway possesses antiviral and antitumor functions. When this pathway is inhibited or inactivated, immune surveillance is weakened. The body becomes unable to effectively sense DNA damage or abnormal DNA, leading to insufficient production of IFN-I, thereby reducing the capacity to eliminate abnormal cells. Long-term inhibition of this pathway may increase the risk of infection as well as the risk of tumorigenesis and metastasis. Therefore, when developing immunomodulatory drugs, strategies such as targeted delivery, intermittent dosing, or local administration should be considered to mitigate the adverse effects of inhibiting this pathway.

## 11 Conclusion

This review focuses on the central role of the cGAS-STING signaling pathway in renal fibrosis, especially in macrophage pro-inflammatory and pro-fibrotic functions. This pathway promotes the release of inflammatory factors and the deposition of extracellular matrix by regulating the polarization states of M1 and M2 macrophages, driving the progression of renal fibrosis. Consequently, targeting the cGAS-STING signaling pathway, including small molecule inhibitors of the cGAS-STING signaling pathway, nanocarrier delivery technologies, and gene editing technologies, provides novel therapeutic strategies for the treatment of renal fibrosis.

Renal fibrosis is mainly characterized by glomerular sclerosis, renal tubular atrophy, and renal interstitial fibrosis. Renal injury can activate various cells, including renal tubular epithelial cells, endothelial cells, fibroblasts, and macrophages. Notably, renal tubular epithelial cells mediate the release of inflammatory factors through the TLR/NF-κB signaling pathway and NLRP3 inflammasome activation. Macrophages critically drive renal fibrosis pathogenesis. Early renal injury establishes an inflammatory microenvironment that promotes monocyte-derived macrophages and M1 polarization, further amplifying the release of inflammatory factors and aggravating renal inflammatory damage. During the repair period, highly expressed M2 macrophages promote tissue repair and inflammation resolution. However, their sustained activation can lead to excessive deposition of ECM components such as collagen and fibronectin, thereby mediating the development of renal fibrosis. Therefore, precisely targeting macrophages is a feasible strategy for treating renal fibrosis.

The cGAS-STING signaling pathway, a crucial core component of the innate immune system, centrally regulates macrophage polarization. On one hand, this pathway promotes M1 polarization, upregulating the expression of pro-inflammatory factors such as TNF-α, IL-1β, and IL-6, thereby exacerbating renal inflammatory responses. Simultaneously, it also drives M2 polarization, accelerating the deposition of collagen, fibronectin, and other ECM depositions, thereby mediating the development of renal fibrosis. Although this mechanism has been extensively studied in various fields such as tumors and autoimmune diseases, its specific role in renal fibrosis remains incompletely defined (75–77). The regulatory effect of the cGAS-STING signaling pathway on macrophages in renal fibrosis provides robust preclinical evidence for the treatment of renal fibrosis.

Pharmacological or genetic inhibition of the cGAS-STING signaling pathway can effectively reprogram the phenotype of macrophages and attenuate renal fibrosis. The current intervention methods mainly include small molecule inhibitors, nanocarrier technology, and gene knockout technology. Small molecule inhibitors targeting the cGAS-STING signaling pathway often include cGAS inhibitors, STING inhibitors, and TBK1 inhibitors. The cGAS inhibitor RU.521 can inhibit the activation of macrophages, suppress the release of pro-inflammatory factors, and reduce the deposition of extracellular matrix. The STING inhibitor C-176 has an essential role in improving renal inflammation and fibrosis. The TBK1 inhibitor H-151 and GSK8612 significantly reduce the transformation of macrophages into fibroblasts, reducing the deposition of ECM. In addition, other emerging technologies are also applied in targeting the cGAS-STING signaling pathway. For example, the AAV9-mediated VBIT-12 vector, STING lentivirus, and the cGAS or STING gene knockout using CRISPR-Cas9 technology inhibit the progression of renal inflammation and fibrosis by targeting the cGAS-STING signaling pathway, providing a novel strategy for precisely targeting macrophages.

However, the translation from current research to clinical application still faces several critical challenges. Although numerous studies have demonstrated that the cGAS-STING pathway plays a significant role in promoting inflammation and fibrosis, its specific effects are context-dependent, characterized by cell-type specificity and disease-stage propensity. This review primarily focuses on the dual role of this pathway in renal macrophages. However, its functions may not be identical in other cell types. For instance, in the liver, activation of this pathway can induce the differentiation of immature dendritic cells into a mature phenotype, upregulating co-stimulatory molecules such as CD80, CD86, and CD11, thereby mediating the proliferation of CD4+ T cells (78). In tumor immunity, the canonical cGAS-STING pathway typically exerts anti-tumor effects by activating CD8+ T cells and inducing the production of IFNs (79). Conversely, the non-canonical STING pathway promotes immune escape by upregulating the secretion of IL-35 and IL-10 in regulatory B cells (Bregs), thereby suppressing the cytotoxic function of NK and CD8+ T cells (80). These studies further substantiate the cell-type specificity of the cGAS-STING signaling pathway.

Secondly, the activation effects of the cGAS-STING pathway may differ across various disease stages. In the early phase of AKI, activation of the cGAS-STING pathway mediated by LPS can promote M1 polarization, thereby initiating inflammatory responses and tissue repair programs. In contrast, chronic kidney injury activates this pathway to promote M2 polarization, subsequently activating fibroblasts and driving the fibrotic process. Notably, the functional status of macrophages is no longer confined to the traditional M1/M2 binary opposition model but instead presents a continuous spectrum model (81). This suggests that the ultimate effects of the cGAS-STING pathway may be highly dependent on the local microenvironment’s cytokine composition and metabolic status. Therefore, the complexity of the pathway’s molecular mechanisms, the specificity of different cell types, and the dynamic diversity of the microenvironment collectively constitute the context-dependent nature and potential contradictions in its functional manifestations. This also represents a major obstacle to its clinical translation.

There is an urgent need to conduct more translational medical research to validate its therapeutic efficacy. Future investigations should employ high-resolution technologies such as single-cell transcriptomics and spatial transcriptomics to deeply explore the dynamic evolution of different macrophage subpopulations at various stages of renal fibrosis and their interactive networks with fibroblasts and renal tubular epithelial cells. This will help elucidate the functional heterogeneity of the cGAS-STING signaling within specific macrophage subsets and provide a theoretical basis for optimizing precision targeted therapeutic strategies. Concurrently, it is essential to systematically evaluate the regulatory role of this signaling pathway in different diseases and its long-term effects in clinical trials, thereby providing a solid theoretical foundation and data support for clinical practice. In conclusion, although the cGAS-STING pathway is still in the exploratory stage for renal fibrosis, its potential in macrophage reprogramming should not be underestimated. By exploring the deeper mechanisms of this pathway in renal fibrosis, it is expected that more effective treatment options will be available for patients with renal fibrosis in the future.

## References

[1] Matsushita K van der Velde M Astor BC Woodward M Levey AS Chronic Kidney Disease Prognosis Consortium . Association of estimated glomerular filtration rate and albuminuria with all-cause and cardiovascular mortality in general population cohorts: a collaborative meta-analysis. Lancet Lond Engl. (2010) 375:2073–81. 20483451 10.1016/S0140-6736(10)60674-5 PMC3993088

[2] Webster AC Nagler EV Morton RL Masson P . Chronic kidney disease. Lancet Lond Engl. (2017) 389:1238–52. doi: 10.1016/S0140-6736(16)32064-5. PMID: 27887750

[3] Xie Y Bowe B Mokdad AH Xian H Yan Y Li T . Analysis of the global burden of disease study highlights the global, regional, and national trends of chronic kidney disease epidemiology from 1990 to 2016. Kidney Int. (2018) 94:567–81. doi: 10.1016/j.kint.2018.04.011. PMID: 30078514

[4] Yan H Xu J Xu Z Yang B Luo P He Q . Defining therapeutic targets for renal fibrosis: exploiting the biology of pathogenesis. BioMed Pharmacother. (2021) 143:112115. doi: 10.1016/j.biopha.2021.112115. PMID: 34488081

[5] Huen SC Cantley LG . Macrophages in renal injury and repair. Annu Rev Physiol. (2017) 79:449–69. doi: 10.1146/annurev-physiol-022516-034219. PMID: 28192060

[6] Onkar PK Ingo H Shrikant RM Jan H Murthy ND Santhosh KV . Toll-like receptor 4-induced IL-22 accelerates kidney regeneration. J Am Soc Nephrol JASN. (2014) 25:978–89. doi: 10.1681/ASN.2013050528. PMID: 24459235 PMC4005301

[7] Thomas AW Kevin MV . Macrophages in tissue repair, regeneration, and fibrosis. Immunity. (2016) 44:450–62. 10.1016/j.immuni.2016.02.015 PMC4794754 26982353

[8] Jiao B An C Du H Tran M Yang D Zhao Y . Genetic deficiency or pharmacological inhibition of cGAS–STING signalling suppresses kidney inflammation and fibrosis. Br J Pharmacol. (2025) 182:1741–62. doi: 10.1111/bph.17412. PMID: 39833988

[9] Chen Q Sun L Chen ZJ . Regulation and function of the cGAS-STING pathway of cytosolic DNA sensing. Nat Immunol. (2016) 17:1142–9. doi: 10.1038/ni.3558. PMID: 27648547

[10] Hopfner KP Hornung V . Molecular mechanisms and cellular functions of cGAS–STING signalling. Nat Rev Mol Cell Biol. (2020) 21:9. doi: 10.1038/s41580-020-0244-x. PMID: 32424334

[11] Patel DJ Yu Y Xie W . cGAMP-activated cGAS–STING signaling: its bacterial origins and evolutionary adaptation by metazoans. Nat Struct Mol Biol. (2023) 30:245–60. doi: 10.1038/s41594-023-00933-9. PMID: 36894694 PMC11749898

[12] Zeng H Gao Y Yu W Liu J Zhong C Su X . Pharmacological inhibition of STING/TBK1 signaling attenuates myeloid fibroblast activation and macrophage to myofibroblast transition in renal fibrosis. Front Pharmacol. (2022) 13:940716. doi: 10.3389/fphar.2022.940716. PMID: 35924048 PMC9340478

[13] Djudjaj S Boor P . Cellular and molecular mechanisms of kidney fibrosis. Mol Aspects Med. (2019) 65:16–36. doi: 10.1016/j.mam.2018.06.002. PMID: 29909119

[14] Lin K Luo W Yang N Su L Zhou H Hu X . Inhibition of MyD88 attenuates angiotensin II-induced hypertensive kidney disease via regulating renal inflammation. Int Immunopharmacol. (2022) 112:109218. doi: 10.1016/j.intimp.2022.109218. PMID: 36116148

[15] Shigeoka AA Kambo A Mathison JC King AJ Hall WF da Silva Correia J . Nod1 and nod2 are expressed in human and murine renal tubular epithelial cells and participate in renal ischemia reperfusion injury. J Immunol Baltim Md 1950. (2010) 184:2297–304. doi: 10.4049/jimmunol.0903065. PMID: 20124104 PMC3020136

[16] Li X Yuan F Xiong Y Tang Y Li Z Ai J . FAM3A plays a key role in protecting against tubular cell pyroptosis and acute kidney injury. Redox Biol. (2024) 74:103225. doi: 10.1016/j.redox.2024.103225. PMID: 38875957 PMC11226986

[17] Sheng L Zhuang S . New insights into the role and mechanism of partial epithelial-mesenchymal transition in kidney fibrosis. Front Physiol. (2020) 11:569322. doi: 10.3389/fphys.2020.569322. PMID: 33041867 PMC7522479

[18] Elson EL Qian H Fee JA Wakatsuki T . A model for positive feedback control of the transformation of fibroblasts to myofibroblasts. Prog Biophys Mol Biol. (2019) 144:30–40. doi: 10.1016/j.pbiomolbio.2018.08.004. PMID: 30174171 PMC11033709

[19] Tomasek JJ Gabbiani G Hinz B Chaponnier C Brown RA . Myofibroblasts and mechano-regulation of connective tissue remodelling. Nat Rev Mol Cell Biol. (2002) 3:349–63. doi: 10.1038/nrm809. PMID: 11988769

[20] Civril F Deimling T De Oliveira Mann CC Ablasser A Moldt M Witte G . Structural mechanism of cytosolic DNA sensing by cGAS. Nature. (2013) 498:332–7. doi: 10.1038/nature12305. PMID: 23722159 PMC3768140

[21] Andreeva L Hiller B Kostrewa D Lässig C De Oliveira Mann CC Jan Drexler D . cGAS senses long and HMGB/TFAM-bound U-turn DNA by forming protein–DNA ladders. Nature. (2017) 549:394–8. doi: 10.1038/nature23890. PMID: 28902841

[22] Ergun SL Ferdandez D Weiss TM Li . STING polymer structure reveals mechanisms for activation, hyperactivation, and inhibition. Cell. (2019) 178:290–301. doi: 10.1016/j.cell.2019.05.036, PMID: 31230712

[23] Zhang Z Zhang C . Regulation of cGAS–STING signalling and its diversity of cellular outcomes. Nat Rev Immunol. (2025) 25:425–44. doi: 10.1038/s41577-024-01112-7. PMID: 39774812

[24] Lu D Shang G Li J Lu Y Bai X Zhang X . Activation of STING by targeting a pocket in the transmembrane domain. Nature. (2022) 604:557–62. doi: 10.1038/s41586-022-04559-7. PMID: 35388221 PMC9098198

[25] Zhang C Shang G Gui X Zhang X Bai X Chen ZJ . Structural basis of STING binding with and phosphorylation by TBK1. Nature. (2019) 567:394–8. doi: 10.1038/s41586-019-1000-2. PMID: 30842653 PMC6862768

[26] Ma C Zhuang J Jia J Li B He Y Huo L . Mitochondria-to-nucleus trafficking NIR-II type I AIE photosensitizer synergistically activates cGAS-STING pathway for efficient tumor eradication. Small. (2025) 21:e03994. doi: 10.1002/smll.202503994. PMID: 40685780

[27] Zhang B Nandakumar R Reinert LS Huang J Laustsen A Gao Z . STEEP mediates STING ER exit and activation of signaling. Nat Immunol. (2020) 21:868–79. doi: 10.1038/s41590-020-0730-5. PMID: 32690950 PMC7610351

[28] Alevtina G Piper T Keith BE Yueh L Michael GJ Glen NB . Autoimmunity initiates in nonhematopoietic cells and progresses via lymphocytes in an interferon-dependent autoimmune disease. Immunity. (2012) 36:120–31. doi: 10.1016/j.immuni.2011.11.018. PMID: 22284419 PMC3269499

[29] Motwani M Pesiridis S Fitzgerald KA . DNA sensing by the cGAS–STING pathway in health and disease. Nat Rev Genet. (2019) 20:657–74. doi: 10.1038/s41576-019-0151-1. PMID: 31358977

[30] Ki WC Poonam D Shizheng H Xin S Rojesh S Chengxiang Q . Mitochondrial damage and activation of the STING pathway lead to renal inflammation and fibrosis. Cell Metab. (2019) 30:784–99. doi: 10.1016/j.cmet.2019.08.003. PMID: 31474566 PMC7054893

[31] Wang J Zhang L Liu Y Liu Y Xiong A Ran Q . Epithelial Atg5 deficiency intensifies Caspase‐11 activation, fueling extracellular mtDNA release to activate cGAS–STING–NLRP3 axis in macrophages during Pseudomonas infection. MedComm. (2025) 6:e70239. doi: 10.1002/mco2.70239. PMID: 40529614 PMC12167704

[32] Kenichi S Timothy RC Justin K Jargalsaikhan D Norika C Shuang C . Oxidized mitochondrial DNA activates the NLRP3 inflammasome during apoptosis. Immunity. (2012) 36:401–14. doi: 10.1016/j.immuni.2012.01.009. PMID: 22342844 PMC3312986

[33] Zhao J Cui M Yao X Jiang Z Qi L Chen J . Targeting TFAM downregulation mediated mtDNA-NLRP3 pathway suppresses TAM infiltration and HCC progression. Oncogene. (2025) 44:2956–69. doi: 10.1038/s41388-025-03467-0. PMID: 40517169

[34] Laura EN Gerald SS . Mitochondrial DNA release in innate immune signaling. Annu Rev Biochem. (2023) 92:299–332. doi: 10.1146/annurev-biochem-032620-104401 . PMID: 37001140 10.1146/annurev-biochem-032620-104401 PMC11058562

[35] Huang H Han Y Zhang Y Zeng J He X Cheng J . Deletion of pyruvate carboxylase in tubular epithelial cell promotes renal fibrosis by regulating SQOR/cGAS/STING‐mediated glycolysis. Adv Sci. (2025) 12:e2408753. doi: 10.1002/advs.202408753. PMID: 39836535 PMC11967762

[36] Po SC Nobuhiro N Hirotoshi E Shingo U Keita S Atsuhiro M . C-C motif chemokine receptor 9 positive macrophages activate hepatic stellate cells and promote liver fibrosis in mice. Hepatol Baltim Md. (2013) 58:337–50. doi: 10.1002/hep.26351. PMID: 23460364

[37] Nikolaos F . Transforming growth factor-β in tissue fibrosis. J Exp Med. (2020) 217:e20190103. doi: 10.1084/jem.20190103. PMID: 32997468 PMC7062524

[38] Paul S Kaplan MH Khanna D McCourt PM Saha AK Tsou PS . Centromere defects, chromosome instability, and cGAS-STING activation in systemic sclerosis. Nat Commun. (2022) 13:7074. doi: 10.1038/s41467-022-34775-8. PMID: 36400785 PMC9674829

[39] Waseem M Imtiaz A Alexander A Graham L Contreras-Galindo R . Crosstalk between oxidative stress, mitochondrial dysfunction, chromosome instability, and the activation of the cGAS-STING/IFN pathway in systemic sclerosis. Ageing Res Rev. (2025) 110:102812. doi: 10.1016/j.arr.2025.102812. PMID: 40562314

[40] Gomes MTR Guimarães ES Marinho FV Macedo I Aguiar ERGR Barber GN . STING regulates metabolic reprogramming in macrophages via HIF-1α during brucella infection. PloS Pathog. (2021) 17:e1009597. doi: 10.1371/journal.ppat.1009597. PMID: 33989349 PMC8153530

[41] Amandeep B Liping H Hong Y Krishna D Steven S Diane LR . Dendritic cell sphingosine 1-phosphate receptor-3 regulates Th1-Th2 polarity in kidney ischemia-reperfusion injury. J Immunol Baltim Md 1950. (2012) 189:2584–96. doi: 10.4049/jimmunol.1200999. PMID: 22855711 PMC3433235

[42] Lee S Huen S Nishio H Nishio S Lee HK Choi BS . Distinct macrophage phenotypes contribute to kidney injury and repair. J Am Soc Nephrol. (2011) 22:317–26. doi: 10.1681/asn.2009060615. PMID: 21289217 PMC3029904

[43] Lech M Gröbmayr R Ryu M Lorenz G Hartter I Mulay SR . Macrophage phenotype controls long-term AKI outcomes—kidney regeneration versus atrophy. J Am Soc Nephrol. (2014) 25:292–304. doi: 10.1681/asn.2013020152. PMID: 24309188 PMC3904561

[44] Julia M Yasunori I Whitney AR Ranu B Yee GY Benjamin DH . CSF-1 signals directly to renal tubular epithelial cells to mediate repair in mice. J Clin Invest. (2009) 119:2330–42. doi: 10.1172/JCI39087. PMID: 19587445 PMC2719924

[45] Ming Z Bing Y Shi Y Li J Su W Xiao F . CSF-1 signaling mediates recovery from acute kidney injury. J Clin Invest. (2012) 122:4519–32. doi: 10.1172/JCI60363. PMID: 23143303 PMC3533529

[46] Feng Y Ren J Gui Y Wei W Shu B Lu Q . Wnt/β-catenin–promoted macrophage alternative activation contributes to kidney fibrosis. J Am Soc Nephrol. (2018) 29:182–93. doi: 10.1681/asn.2017040391. PMID: 29021383 PMC5748914

[47] Niehrs C . The complex world of WNT receptor signalling. Nat Rev Mol Cell Biol. (2012) 13:767–79. doi: 10.1038/nrm3470. PMID: 23151663

[48] Zhu QQ Ma C Wang Q Song Y Lv T . The role of TWIST1 in epithelial-mesenchymal transition and cancers. Tumor Biol. (2016) 37:185–97. doi: 10.1007/s13277-015-4450-7. PMID: 26602382

[49] Wu Q Sun S Wei L Liu M Liu H Liu T . Twist1 regulates macrophage plasticity to promote renal fibrosis through galectin-3. Cell Mol Life Sci. (2022) 79:137. doi: 10.1007/s00018-022-04137-0. PMID: 35182235 PMC8858306

[50] Ying L Xiao L Lu Y Lei W Zhao S Xiu G . Roles of galectin-3 in metabolic disorders and tumor cell metabolism. Int J Biol Macromol. (2020) 142:463–73. doi: 10.1016/j.ijbiomac.2019.09.118. PMID: 31604080

[51] Nørgård MØ Christensen M Mutsaers HAM Nørregaard R . Phenformin attenuates renal injury in unilateral ureteral obstructed mice without affecting immune cell infiltration. Pharmaceutics. (2020) 12:301. doi: 10.3390/pharmaceutics12040301. PMID: 32224876 PMC7238166

[52] Pepermans RA Sharma G Prossnitz ER . G protein-coupled estrogen receptor in cancer and stromal cells: Functions and novel therapeutic perspectives. Cells. (2021) 10:672. doi: 10.3390/cells10030672. PMID: 33802978 PMC8002620

[53] Rettew JA McCall SH Marriott I . GPR30/GPER-1 mediates rapid decreases in TLR4 expression on murine macrophages. Mol Cell Endocrinol. (2010) 328:87–92. doi: 10.1016/j.mce.2010.07.017. PMID: 20654686

[54] Wei T Chen W Wen L Zhang J Zhang Q Yang J . G protein-coupled estrogen receptor deficiency accelerates liver tumorigenesis by enhancing inflammation and fibrosis. Cancer Lett. (2016) 382:195–202. doi: 10.1016/j.canlet.2016.08.012. PMID: 27594673

[55] Xie L Cheng Y Du W Fu L Wei Z Guan Y . Activation of GPER1 in macrophages ameliorates UUO-induced renal fibrosis. Cell Death Dis. (2023) 14:818. doi: 10.1038/s41419-023-06338-2. PMID: 38086848 PMC10716282

[56] Ni J Guo T Zhou Y Jiang S Zhang L Zhu Z . STING signaling activation modulates macrophage polarization via CCL2 in radiation-induced lung injury. J Transl Med. (2023) 21:590. doi: 10.1186/s12967-023-04446-3. PMID: 37667317 PMC10476398

[57] Luo X Li H Ma L Zhou J Guo X Woo SL . Expression of STING is increased in liver tissues from patients with NAFLD and promotes macrophage-mediated hepatic inflammation and fibrosis in mice. Gastroenterology. (2018) 155:1971–1984.e4. doi: 10.1053/j.gastro.2018.09.010. PMID: 30213555 PMC6279491

[58] Ji T Zhao TT Long SZ Wei CZ Cheng DY Chen J . Microvesicle-transferred mitochondria trigger cGAS-STING and reprogram metabolism of macrophages in sepsis. Microbiol Spectr. (2025) 13:e0078125. doi: 10.1128/spectrum.00781-25. PMID: 40905697 PMC12502667

[59] Sun J Sun Y Guo D Ye H Huang Q Zhou H . Targeting STING to disrupt macrophage-mediated adhesion in encapsulating peritoneal sclerosis. Commun Biol. (2025) 8:1266. doi: 10.1038/s42003-025-08662-z. PMID: 40847090 PMC12373792

[60] Zhao J Xiao R Zeng R He E Zhang A . Small molecules targeting cGAS-STING pathway for autoimmune disease. Eur J Med Chem. (2022) 238:114480. doi: 10.1016/j.ejmech.2022.114480. PMID: 35635952

[61] Xu Q Xiong H Zhu W Liu Y Du Y . Small molecule inhibition of cyclic GMP-AMP synthase ameliorates sepsis-induced cardiac dysfunction in mice. Life Sci. (2020) 260:118315. doi: 10.1016/j.lfs.2020.118315. PMID: 32835697

[62] Haag SM Gulen MF Reymond L Gibelin A Abrami L Decout A . Targeting STING with covalent small-molecule inhibitors. Nature. (2018) 559:269–73. doi: 10.1038/s41586-018-0287-8. PMID: 29973723

[63] Hu S Gao Y Gao R Wang Y Qu Y Yang J . The selective STING inhibitor H-151 preserves myocardial function and ameliorates cardiac fibrosis in murine myocardial infarction. Int Immunopharmacol. (2022) 107:108658. doi: 10.1016/j.intimp.2022.108658, PMID: 35278833

[64] Kim HJ Kim HJ Kim SY Roh J Yun JH Kim CH . TBK1 is a signaling hub in coordinating stress-adaptive mechanisms in head and neck cancer progression. Autophagy. (2025) 21:1744–66. doi: 10.1080/15548627.2025.2481661, PMID: 40114316 PMC12282999

[65] Liu Z Qin Q Wang S Kang X Liu Y Wei L . STING Activation in Macrophages and Microglia Drives Poststroke Inflammation: Implications for Neuroinflammatory Mechanisms and Therapeutic Interventions. CNS Neurosci Ther. (2024) 30:e70106. doi: 10.1111/cns.70106, PMID: 39698742 PMC11656094

[66] Xian H Watari K Sanchez-Lopez E Offenberger J Onyuru J Sampath H . Oxidized DNA fragments exit mitochondria via mPTP- and VDAC-dependent channels to activate NLRP3 inflammasome and interferon signaling. Immunity. (2022) 55:1370–1385.e8. doi: 10.1016/j.immuni.2022.06.007, PMID: 35835107 PMC9378606

[67] Hu H Guo L Overholser J Wang X . Mitochondrial VDAC1: A potential therapeutic target of inflammation-related diseases and clinical opportunities. Cells. (2022) 11:3174. doi: 10.3390/cells11193174, PMID: 36231136 PMC9562648

[68] Wang D Li Y Li G Liu M Zhou Z Wu M . Inhibition of PKC-d retards kidney fibrosis via inhibiting cGAS-STING signaling pathway in mice. Cell Death Discov. (2024) 10:314. doi: 10.1038/s41420-024-02087-z, PMID: 38972937 PMC11228024

[69] Zhang C Ye S Ni J Cai T Liu Y Huang D . STING signaling remodels the tumor microenvironment by antagonizing myeloid-derived suppressor cell expansion. Cell Death Differ. (2019) 26:2314–28. doi: 10.1038/s41418-019-0302-0, PMID: 30816302 PMC6889506

[70] Jiang A Liu J Wang Y Zhang C . cGAS-STING signaling pathway promotes hypoxia-induced renal fibrosis by regulating PFKFB3-mediated glycolysis. Free Radic Biol Med. (2023) 208:516–29. doi: 10.1016/j.freeradbiomed.2023.09.011, PMID: 37714438

[71] Ha MH Kim MS An H Sung M Lee YH Yang D . PTEN‐induced kinase 1 is associated with renal aging, via the cGAS‐STING pathway. Aging Cell. (2023) 22:e13865. doi: 10.1111/acel.13865, PMID: 37183600 PMC10352563

[72] Gairola S Kaundal RK . Amlexanox alleviates renal inflammation and fibrosis by inhibiting cGAS/STING/TBK1 and TGF-β1/smad signaling. Eur J Pharmacol. (2025) 1007:178266. doi: 10.1016/j.ejphar.2025.178266, PMID: 41110750

[73] Chen B Rao X Wang X Luo Z Wang J Sheng S . cGAS-STING signaling pathway and liver disease: From basic research to clinical practice. Front Pharmacol. (2021) 12:719644. doi: 10.3389/fphar.2021.719644, PMID: 34483930 PMC8416453

[74] Zhu Z Zhou X Du H Cloer EW Zhang J Mei L . STING suppresses mitochondrial VDAC2 to govern RCC growth independent of innate immunity. Adv Sci. (2022) 10:2203718. doi: 10.1002/advs.202203718, PMID: 36445063 PMC9875608

[75] Wu Y Fang Y Wei Q Shi H Tan H Deng Y . Tumor-targeted delivery of a STING agonist improves cancer immunotherapy. Proc Natl Acad Sci. (2022) 119:e2214278119. doi: 10.1073/pnas.2214278119, PMID: 36442099 PMC9894229

[76] Zhang W Huang X . Targeting cGAS-STING pathway for reprogramming tumor-associated macrophages to enhance anti-tumor immunotherapy. Biomark Res. (2025) 13:43. doi: 10.1186/s40364-025-00750-w, PMID: 40075527 PMC11905658

[77] Anees F Montoya DA Pisetsky DS Payne CK . DNA corona on nanoparticles leads to an enhanced immunostimulatory effect with implications for autoimmune diseases. Proc Natl Acad Sci. (2024) 121:e2319634121. doi: 10.1073/pnas.2319634121, PMID: 38442162 PMC10945806

[78] Ren C Cui X Wang H Jin C Gao L Li Y . In virto priming of the STING signaling pathway enhances the maturation and activation of dendritic cells induced by hepatitis B vaccine. Immunol Lett. (2025) 272:106977. doi: 10.1016/j.imlet.2025.106977, PMID: 39921064

[79] Al-janabi H Moyes K Allen R Fisher M Crespo M Gurel B . Targeting a STING agonist to perivascular macrophages in prostate tumors delays resistance to androgen deprivation therapy. J Immunother Cancer. (2024) 12:e009368. doi: 10.1136/jitc-2024-009368, PMID: 39060021 PMC11284826

[80] Li S Mirlekar B Johnson BM Brickey WJ Wrobel JA Yang N . STING-induced regulatory B cells compromise NK function in cancer immunity. Nature. (2022) 610:373–80. doi: 10.1038/s41586-022-05254-3, PMID: 36198789 PMC9875944

[81] Cheung MD Erman EN Moore KH Lever JM Li Z LaFontaine JR . Resident macrophage subpopulations occupy distinct microenvironments in the kidney. JCI Insight. (2022) 7:e161078. doi: 10.1172/jci.insight.161078, PMID: 36066976 PMC9714795

